# TNFSF14 (LIGHT) Exhibits Inflammatory Activities in Lung Fibroblasts Complementary to IL-13 and TGF-β

**DOI:** 10.3389/fimmu.2018.00576

**Published:** 2018-03-19

**Authors:** Ricardo da Silva Antunes, Amit K. Mehta, Lisa Madge, Joel Tocker, Michael Croft

**Affiliations:** ^1^Division of Immune Regulation, La Jolla Institute for Allergy and Immunology, La Jolla, CA, United States; ^2^Janssen Research and Development, LLC, Immunology Discovery Research, Spring House, PA, United States; ^3^Department of Medicine, University of California San Diego, La Jolla, CA, United States

**Keywords:** asthma, lung fibroblasts, homologous to Lymphotoxin, exhibits Inducible expression and competes with HSV Glycoprotein D for binding to HVEM, a receptor expressed on T lymphocytes, IL-13, TGF-β

## Abstract

The cytokine TNFSF14 [homologous to Lymphotoxin, exhibits Inducible expression and competes with HSV Glycoprotein D for binding to HVEM, a receptor expressed on T lymphocytes (LIGHT)] has been shown in mouse models to be important for development of lung tissue remodeling that is characteristic of asthma, idiopathic pulmonary fibrosis (IPF), and systemic sclerosis (SSc). However, its cellular targets are not fully delineated. In the present report, we show that LTβR and HVEM, the receptors for LIGHT, are constitutively expressed in primary human lung fibroblasts (HLFs). We asked whether LIGHT could promote inflammatory and remodeling-relevant activity in HLFs and how this was similar to, or distinct from, IL-13 or TGF-β, two cytokines strongly implicated in the pathogenesis of asthma, IPF, and SSc. Accumulation of myofibroblasts expressing alpha smooth muscle actin is a feature of lung inflammatory diseases. LIGHT promoted cell cycle progression and proliferation of HLFs, but not alpha smooth muscle actin expression. In contrast, TGF-β upregulated alpha smooth muscle actin but did not drive their proliferation. LIGHT also increased the gene or protein expression of a number of proinflammatory mediators, including ICAM-1 and VCAM-1, IL-6 and GM-CSF, the chemokines CCL5 and 20, and CXCL5, 11, and 12, and lung remodeling-associated proteinases MMP-9 and ADAM8. These were dependent on LTβR but not HVEM. LIGHT displayed overlapping and synergistic activities with IL-13 for a number of the activities, but LIGHT additionally enhanced the gene expression of several molecules, including the innate cytokines IL-33 and TSLP, which were not upregulated by IL-13. Our results highlight the varied and pleiotropic effects of LIGHT in HLFs. LIGHT might then be a therapeutic target for modulation of inflammation and remodeling associated with asthma and other similar diseases of the lung that involve fibroblasts.

## Introduction

Lung fibroblasts can play a key role in orchestration of immune responses and tissue remodeling that are characteristic of lung inflammatory diseases. Initially regarded solely as a structure cell, the fibroblast is now considered of primary importance to disease pathogenesis in populations of patients that exhibit severe asthma, idiopathic pulmonary fibrosis (IPF), and the interstitial lung disease found in systemic sclerosis (SSc). In this regard, the accumulation of higher than normal numbers of fibroblasts can impair the usual elasticity of the lungs. Their upregulation of alpha smooth muscle actin and differentiation into the myofibroblast phenotype, with contractile characteristics of smooth muscle cells, further contributes to poor functioning of the airways. Additionally, lung fibroblasts can produce a plethora of inflammatory and tissue remodeling factors. These include extracellular matrix proteins such as collagen, proteinase enzymes that modify the extracellular matrix as well as enhance the availability of inflammatory molecules, and chemokines that attract inflammatory immune cells (1–4).

Although the role of the lung fibroblast in disease is established, the most important molecules that regulate lung fibroblast activity are not known. A number of studies have described strong effects of the immune cell-derived cytokines TGF-β and IL-13 in contributing to various aspects of fibroblast growth or differentiation (5–10). However, it remains unclear how many other inflammatory molecules might promote fibroblast activity that is associated with the lung diseases that have a fibrotic component.

In this regard, we previously implicated the TNF superfamily molecule homologous to Lymphotoxin, exhibits Inducible expression and competes with HSV Glycoprotein D for binding to HVEM, a receptor expressed on T lymphocytes (LIGHT) (aka CD235/TNFSF14) in the development of lung fibrosis and tissue remodeling. We showed that mice lacking LIGHT or treated with a LIGHT-neutralizing reagent are strongly protected from exhibiting these features in models of asthma and SSc (11, 12). Like IL-13, LIGHT is a primary product of T cells (13), and it can exert activities on a number of immune cells, including T cells, macrophages, and eosinophils, that can be present or recruited into the lungs (11, 14). Additionally, we recently found that its two receptors, LTβR and HVEM, were expressed in human bronchial epithelial (HBE) cells (12, 15), and that LIGHT through binding LTβR induced a range of inflammatory and remodeling-relevant activities in these structural cells (12, 15). This then led to speculation that LIGHT could display similar or additional functions in other lung structural cells, including fibroblasts (16).

Here, we show that LIGHT promoted proliferation and increased cell cycle progression of human lung fibroblasts (HLFs), albeit with no significant activity in upregulating alpha smooth muscle actin expression. This contrasted with TGF-β that inhibited fibroblast proliferation but induced alpha smooth muscle actin. LIGHT stimulation also induced a limited but defined set of genes, including adhesion molecules, innate cytokines, chemokines, and proteinases, that have been implicated in inflammation and remodeling in the lungs. Importantly, LIGHT displayed similar activity to IL-13 in lung fibroblasts with respect to upregulation of several of the inflammatory genes, as well as distinct activity, including the induction of gene transcription for the innate cytokines IL-33 and TSLP which were not promoted by IL-13. Thus, LIGHT signaling can promote a specific inflammatory signature in lung fibroblasts that is complementary yet distinct from TGF-β and IL-13, two molecules thought to be central to lung fibrosis and dysregulation seen in asthma, IPF, or SSc.

## Materials and Methods

### Cells

Primary normal HLF were purchased from Lonza (Walkersville, MD, USA) and maintained in fibroblast basal medium supplemented with cytokines and growth factors and 2% serum (FGM-2 bulletkit, Lonza). Primary HBE cells were from Lonza and maintained in bronchial epithelial basal medium supplemented with cytokines and growth factors (BEGM, Lonza). Cells (0.5–1 × 10^6^) were grown as monolayers and cultured until about 90–95% confluent, then treated with 0.05% solution of Trypsin/EDTA, washed with HBSS (Corning, Manassas, VA, USA), and resuspended in medium for experiments. Cells were used between passages 2–5 for HLF and 2–3 for HBE. Cells from several donors were used for reproducibility.

### Reagents and Flow Cytometry Antibodies

Trypsin/EDTA and antibiotic/antimycotic solution (penicillin/streptomycin, thereafter P/S) were from Life Technologies (Carlsbad, CA, USA). Fetal bovine serum was from Omega Scientific (Tarzana, CA, USA). Propidium iodide (PI) was from Sigma (St. Louis, MO, USA). Recombinant human LIGHT/TNFSF14, lymphotoxin α1/β2, TGF-β1, BTLA-Fc chimera protein, IL-13, and human antibody to MMP-9 were obtained from R&D Systems (Minneapolis, MN, USA). Antihuman CD270 (HVEM clone eBioHVEM-122) and CD54 (ICAM-1, clone HA58) were from eBioscience (San Diego, CA, USA). Anti-human CD106 (VCAM-1, clone STA) and LTβR (clone 31G4D8) were from BioLegend (San Diego, CA, USA). Antibodies to IL-4Rα and IL-13Rα were from R&D. Flow cytometry staining was as described previously (15). Isotype matched mAb were used as negative controls. Cells were acquired with a FACSCalibur (Becton Dickinson, Mountain View, CA, USA) and analyzed using FlowJo software.

### Culture Conditions

Human lung fibroblast or HBE were seeded a day prior to starting the treatment in 6 well plates (Becton Dickinson, Mountain View, CA, USA) at a density of 0.05–0.25 × 10^6^/ml in 2 ml. Cells were cultured alone, or with recombinant LIGHT/TNFSF14 (6–100 ng/ml), recombinant lymphotoxin α1/β2 (50 ng/ml), BTLA-Fc (500 ng/ml), recombinant IL-13 (25 ng/ml), or TGF-β1 (0.1–5 ng/ml) for 1–3 days. Cells were generally cultured for 48 hrs for mRNA analysis, a time point found to be optimal for LIGHT induction of target genes. In some cases, cells were stimulated with a membrane form of LIGHT present on the surface of a transfected cell line (EL4-LIGHT). Fibroblasts stimulated with membrane-bound LIGHT displayed similar responses compared to those stimulated with soluble LIGHT (not shown). HBE were stimulated in submerged cultures based on initial experiments showing similar results in air–liquid interface cultures.

### Determination of Proliferation

Fibroblasts were cultured for 3 days and proliferation was studied by two methods: (1) thymidine uptake and (2) evaluation of DNA content. For thymidine uptake, 0.5 μCi [3H]-TdR (specific activity, 5.0 Ci/mmol; PerkinElmer, Waltham, MA, USA) was added 16 h before the end of the culture, and cells were harvested on MicroBeta FilterMate semi-automatic cell harvester (PerkinElmer). The incorporated [3H]-TdR was measured in a Beckman liquid scintillation counter, and results were expressed as counts per minute. To evaluate DNA content, cells were permeabilized with ice-cold 70% ethanol for 10 min. Cells were then washed with PBS and stained for 30 min at 37°C with 50 µg/ml of PI. Labeled cells were immediately acquired in a FACScalibur and DNA content (PI fluorescence) monitored on the FL2 channel.

### RNA Isolation and qRT-PCR

RNA isolation and real time PCR analysis was performed as before (15) using specific primers (IDT, Coralville, IA, USA) already described (15). PCR products were generated in triplicate and normalized to housekeeping genes, β-actin or GAPDH. Relative quantification (RQ) was derived from the difference in cycle threshold (Ct) between the gene of interest and the housekeeping genes using the equation RQ = 2^−ΔΔct^. Data in each experiment are displayed as the average RQ or fold increase above baseline expression.

### Transfection of siRNA

ON-TARGETplus siRNA were purchased from Dharmacon (Pittsburg, PA, USA). 5–25 nM of control, HVEM, or LTβR siRNA were transfected into fibroblasts using HiPerFect transfection reagent (Qiagen, Venlo, Netherlands) according to the manufacturer’s instructions. Knockdown was monitored by mRNA and surface protein levels and was stable during the 48hr culture period with LIGHT.

### Measurement of Soluble Proteins and Immunoblotting

Supernatants from stimulated HLF or HBE were centrifuged at 13,000 *g* for 15 min and stored at −80°C until used. The levels of IL-6, GM-CSF, CCL5, CXCL11 and CXCL12, TSLP, IL-33, ADAM8, MMP-9, and CCL26 were determined with ELISA-based kits (R&D Systems), according to the manufacturer’s instructions. MMP-9 immunoblotting was performed as before (15). Briefly, supernatants of treated cells were precipitated with TCA, resuspended in sample buffer and resolved in polyacrylamide gels under reducing conditions, then transferred to PVDF membranes and incubated with anti-MMP-9 followed by horseradish peroxidase-conjugated secondary Ab.

### Statistical Analysis

Statistical analyses were performed using Excel or GraphPad Prism 6 software. A two-tail unpaired Student’s *t-*test between two groups, or one-way ANOVA with Greenhouse-Geisser correction followed by Dunnett’s multiple comparisons test, was used to test the significance of the differences between group means. All data are representative of at least three independent experiments with different donor cell populations and expressed as mean ± SEM. Statistical significance was defined as *P < 0.05.

## Results

### HLFs Express LTβR and HVEM and Proliferate and Upregulate Adhesion Molecules in Response to LIGHT

**Figure 1:**
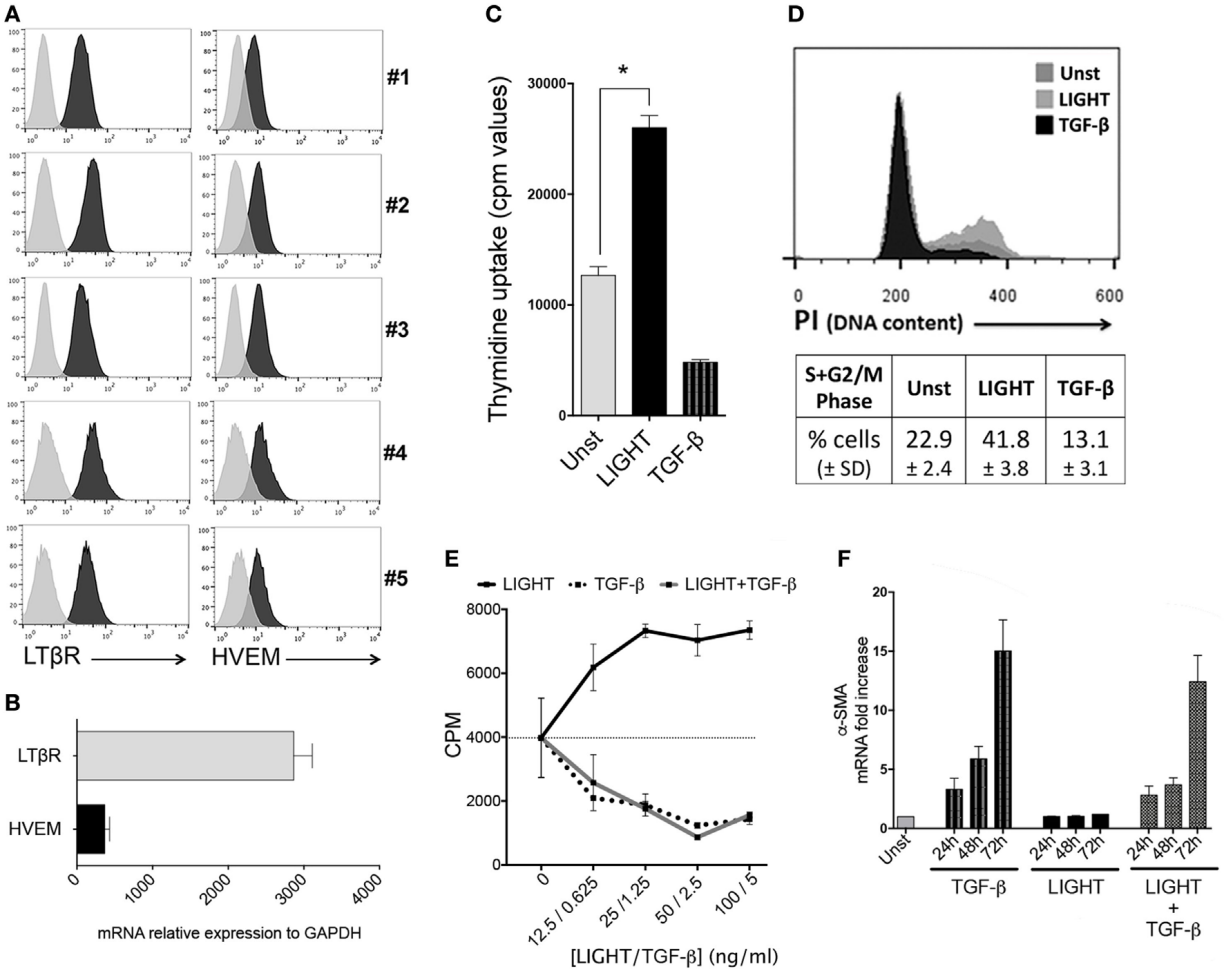
Homologous to Lymphotoxin, exhibits Inducible expression and competes with HSV Glycoprotein D for binding to HVEM, a receptor expressed on T lymphocytes (LIGHT) promotes proliferation of lung fibroblasts. **(A)** Primary human lung fibroblasts (HLF) from five individual donors were assessed by flow cytometry analysis for expression of LTβR and HVEM. Isotype control, gray line. **(B)** Human lung fibroblast (HLF) mRNA for LTβR and HVEM. Data are mean ± SEM from two donors. **(C,D)** Proliferation of HLF was assessed after 3 days when cultured in the absence (Unst) or presence of soluble recombinant LIGHT (50 ng/ml) or TGF-β (5 ng/ml). **(C)** Uptake of tritiated thymidine. **(D)** Progression into S and G2/M phases of the cell cycle by DNA content [propidium iodide (PI)] analysis. Data are mean ± SEM of triplicate cultures from three independent experiments. Similar data obtained from a second donor population. **(E)** Proliferation of HLF to varying doses of LIGHT (12.5–100 ng/ml) or TGF-β (0.6–5 ng/ml) alone, or 100 ng/ml LIGHT combined with varying doses of TGF-β. Data are mean ± SEM of quadruplicate cultures. **(F)** Kinetics of α-SMA mRNA expression in HLF over 3 days when cultured in the absence (Unst) or presence of soluble recombinant LIGHT (50 ng/ml) or TGF-β (5 ng/ml) or both cytokines. Data expressed as fold increase relative to unstimulated. Data are mean ± SEM of two donors and representative of four experiments.

We examined the expression of LTβR and HVEM, the receptors for LIGHT, in five normal HLF cultures obtained from individual donors purchased from Lonza. All HLF cells expressed constitutively high levels of LTβR. HVEM was also constitutively expressed but at markedly lower levels, confirmed by flow cytometry as well as PCR (Figures 1A,B). The pattern of expression of both receptors was similar between donors, suggesting that HLF could be highly responsive to LIGHT.

Our previous data from *in vivo* studies of murine models of severe asthma (11) and SSc (12) showed that LIGHT promoted an increase in lung smooth muscle mass, in a manner partially dependent on TGF-β. This might have been due to hyperplasia of mature smooth muscle as well as accumulation of fibroblasts expressing alpha smooth muscle actin. We then first tested whether LIGHT could promote the division of HLF, relevant to the latter activity. As shown in (Figure 1C), treatment with soluble recombinant LIGHT significantly enhanced uptake of tritiated thymidine by HLF as well as induced greater cell cycle progression into S and G2/M phases as measured by PI staining (Figure 1D). This response was dose dependent, with maximal activity at 25–100 ng/ml of the soluble molecule (Figure 1E).

Previous data have shown that TGF-β can act on fibroblasts. However, in contrast to the positive effect of LIGHT on HLF division, TGF-β blocked the proliferation of these cells when cultured alone as well as when cocultured with LIGHT (Figures 1C–E). TGF-β on the other hand has been reported to promote alpha smooth muscle actin expression in HLF (9, 17). We confirmed alpha smooth muscle actin induction by TGF-β but found that LIGHT did not significantly promote this feature alone, or enhance the activity of TGF-β. In fact, LIGHT had a moderate negative effect on the ability of TGF-β to promote alpha smooth muscle actin, although this somewhat dependent on the time analyzed or dose of TGF-β used (Figure 1F; Figure S1 in Supplementary Material). These results suggest that LIGHT and TGF-β have distinct actions, but may work together albeit at different times, to enhance the accumulation of greater numbers of lung fibroblasts that express alpha smooth muscle actin.

**Figure 2:**
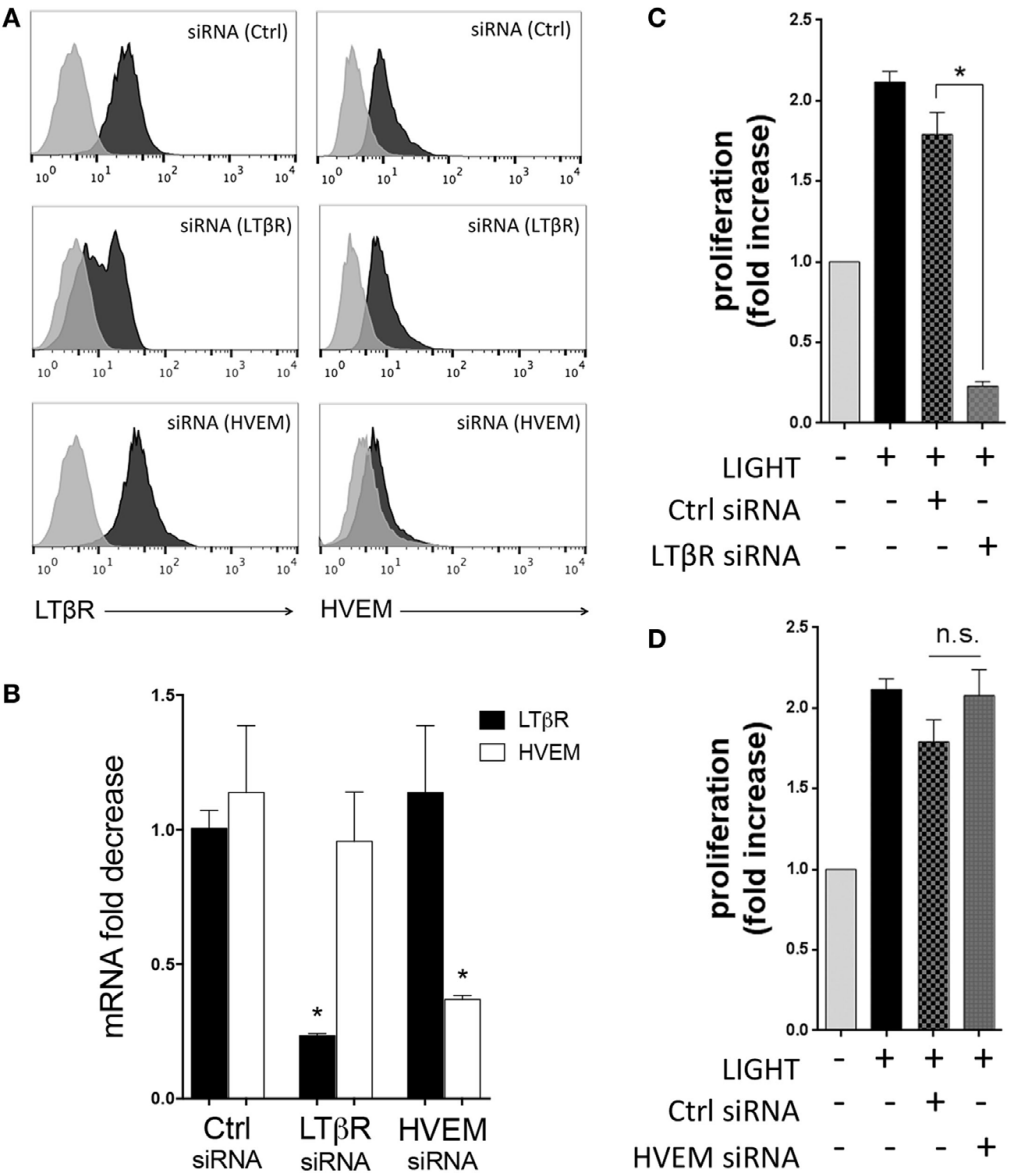
Homologous to Lymphotoxin, exhibits Inducible expression and competes with HSV Glycoprotein D for binding to HVEM, a receptor expressed on T lymphocytes (LIGHT) induces lung fibroblast proliferation through LTβR. Human lung fibroblasts (HLF) were transfected with control and siRNA against LTβR or HVEM. Flow cytometry **(A)** and mRNA analysis **(B)** demonstrating the extent of knockdown of each molecule. **(C,D)** HLF with siRNA knockdown of LTβR **(C)** and HVEM **(D)** were stimulated with LIGHT (50 ng/ml) and proliferation assessed by thymidine incorporation. Data expressed as fold increase relative to unstimulated. Data are mean ± SEM of two donors and representative of four experiments.

In previous studies, we found that LIGHT stimulation of HBE cells resulted in endocytosis or cleavage of LTβR from the membrane, indicative of active signaling through this receptor (15). Similarly, exposure of HLF to LIGHT resulted in downregulation of surface LTβR but not HVEM (data not shown), implying that LTβR might be the primary receptor active on these cells. To investigate the contributions of HVEM and LTβR to cell division, HLF were then transfected with siRNA against these molecules. Flow cytometry and mRNA expression confirmed strong downregulation of both receptors with no effect on the non-targeted receptor (Figures 2A,B). This revealed that LIGHT-induced proliferation was dependent on LTβR but not HVEM (Figures 2C,D).

**Figure 3:**
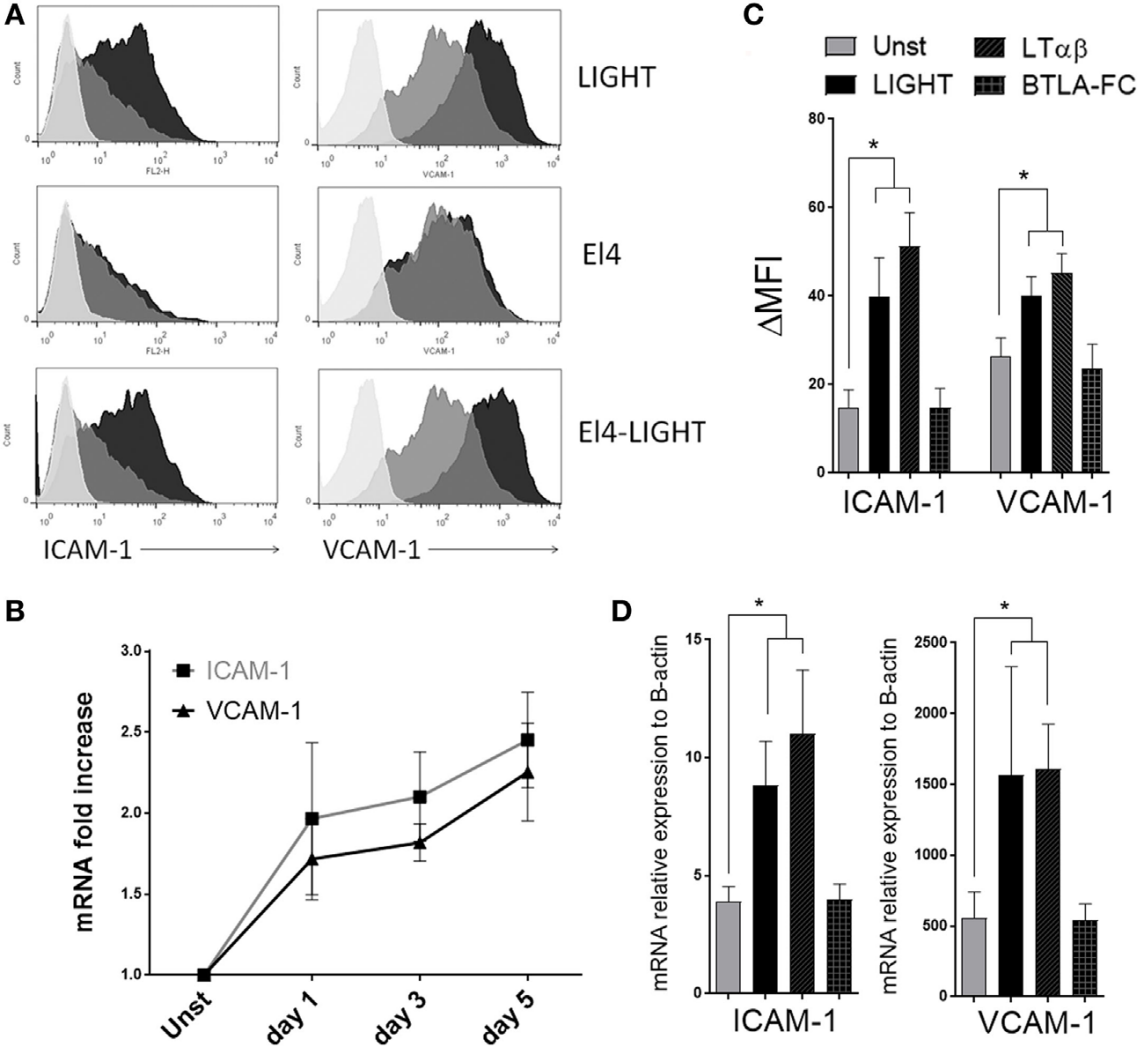
Homologous to Lymphotoxin, exhibits Inducible expression and competes with HSV Glycoprotein D for binding to HVEM, a receptor expressed on T lymphocytes (LIGHT) upregulates ICAM-1 and VCAM-1 in lung fibroblasts. Human lung fibroblasts (HLF) were cultured in the absence or presence of soluble recombinant LIGHT (50 ng/ml), or LIGHT expressed in the membrane of EL4 cells (EL4-LIGHT). **(A)** ICAM-1 and VCAM-1 expression in representative cells assessed by flow cytometry at 48 h. Unstimulated (gray); experimental conditions (black). Isotype control, light gray. **(B)** Mean mRNA fold increase above unstimulated over 5 days with soluble LIGHT. Data from four donors. **(C,D)** HLF were cultured with soluble LIGHT, LTαβ, or BTLA-Fc, and MFI **(C)** and mRNA expression relative to actin **(D)** of ICAM-1 and VCAM-1 assessed at 48 h. Data from three donors and representative of three independent experiments.

We also reported that LIGHT can augment the expression of adhesion molecules in bronchial epithelial cells (15). As this type of activity might also be important for the accumulation of fibroblasts in the lungs, either affecting their migration within the lung and/or maintenance around the sub-bronchiolar epithelial regions, we examined whether LIGHT could promote expression of ICAM-1 and VCAM-1 in HLF. All HLF constitutively expressed high levels of VCAM-1 and lower levels of ICAM-1. Importantly, soluble LIGHT upregulated both VCAM-1 and ICAM-1, in a time-dependent manner, at both the mRNA and protein levels (Figures 3A,B). LIGHT can be naturally expressed by T cells and other immune cells as a cell surface molecule or soluble after being cleaved from the membrane. We therefore compared soluble recombinant LIGHT to a membrane form of LIGHT that cannot be cleaved that was presented on the surface of a transfected cell line (EL4-LIGHT). Showing a similar activity, membrane LIGHT also strongly enhanced expression of both VCAM-1 and ICAM-1 (Figure 3A).

As an alternative way to investigate the contributions of HVEM and LTβR to the LIGHT-induced response, we stimulated HLF with either LTαβ or BTLA-Fc that signal through LTβR and HVEM, respectively. Confirming the prior data, after LTαβ stimulation the levels of both ICAM-1 and VCAM-1 were similar to those in LIGHT-treated HLF at both protein and mRNA levels. In contrast, no upregulation was observed with BTLA-Fc even though BTLA-Fc was able to bind HVEM (Figures 3C,D and data not shown). Therefore, LIGHT promotes several activities relevant to accumulation of fibroblasts in the lungs, mediated by signaling through LTβR.

### LIGHT Induces a Specific Profile of Proinflammatory Mediators in Lung Fibroblasts

**Figure 4:**
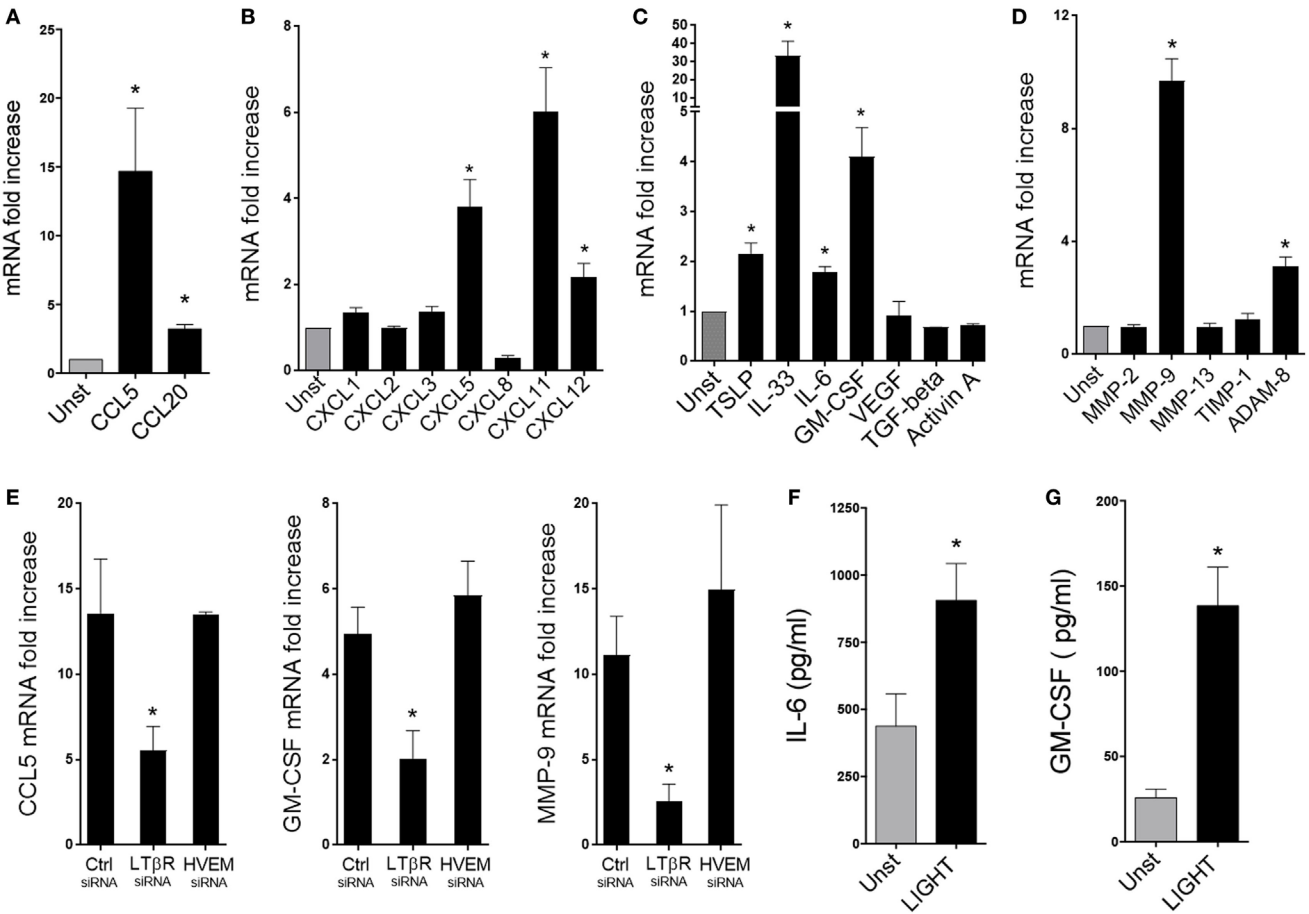
Homologous to Lymphotoxin, exhibits Inducible expression and competes with HSV Glycoprotein D for binding to HVEM, a receptor expressed on T lymphocytes (LIGHT) upregulates the expression of a select group of chemokines, cytokines, and proteinases in lung fibroblasts. Human lung fibroblasts (HLFs) were left unstimulated (gray bars) or stimulated for 48 h with 50 ng/ml soluble recombinant LIGHT (black bars). **(A–E)** mRNA fold increase of the indicated molecules. **(E)** Induction of mRNA for CCL5, GM-CSF, and MMP-9 in HLF with siRNA knockdown of LTβR or HVEM. All data are mean ± SEM from triplicate cultures from three independent experiments, shown as fold increase above unstimulated. **(F,G)** Mean IL-6 **(F)** and GM-CSF **(G)** protein levels in HLF supernatants after 3 days. *n* = 5 independent experiments.

Fibroblasts can also produce chemokines and other proinflammatory mediators that contribute to lung inflammation and tissue remodeling in asthma and other similar diseases. We therefore assessed the contribution of LIGHT to several of these activities in HLF. Treatment with soluble LIGHT specifically enhanced gene expression of CCL5, and to a lesser extent CCL20, major chemotactic factors for T cells, eosinophils, and basophils (Figure 4A). In contrast, other CC chemokine genes, like CCL2, CCL3, CCL17, CCL21, and CCL22, chemoattractants for monocytes/macrophages, and CCL11, CCL24, and CCL26, chemoattractants for eosinophils, were not upregulated after LIGHT stimulation (data not shown). Also, we found that LIGHT enhanced transcription of the genes for CXCL5 and CXCL11, potent chemoattractants for lymphocytes and neutrophils, and CXCL12, that aids chemotaxis of neutrophils and lymphocytes and synergizes with other chemokines (Figure 4B). Other CXC family chemokine genes like CXCL1, CXCL2, CXCL3, and CXCL8 were not induced by LIGHT (Figure 4B). CXCL10 was not expressed in unstimulated HLF, and LIGHT also did not induce transcription of this chemokine (data not shown).

Furthermore, we found that LIGHT promoted increased gene expression of innate cytokines that are implicated in activation of ILC2s as well as other cell types, with transcription of IL-33 being strongly induced and TSLP elevated to a lesser extent (Figure 4C). Gene expression of the growth/differentiation factors IL-6 and GM-CSF was additionally enhanced by LIGHT. In contrast, genes expression of VEGF, TGF-β, and Activin A, that have also been implicated in lung inflammatory and remodeling responses, were unaffected (Figure 4C). Moreover, we found a selective activity of LIGHT in enhancing transcription of MMP-9 and ADAM8, two molecules implicated in modulating extracellular matrix deposition in the lungs. No effect was seen on the metalloproteinase genes, MMP-2 and -13, or the tissue inhibitor of metalloproteinases TIMP-1 (Figure 4D). Interestingly, LIGHT-induced gene expression was again significantly decreased with siRNA to LTβR, but not siRNA to HVEM (Figure 4E and not shown). As with proliferation, maximal activity of LIGHT in promoting the above factors was generally seen at doses of 25–100 ng/ml (Figure S2 in Supplementary Material). Assessing the kinetics of gene transcription showed that LIGHT activity was evident within 24 h and either maintained or increased over 72 h, and that differential kinetics was unlikely to explain why LIGHT did not promote expression of certain genes (Figure S3 in Supplementary Material).

These results emphasize that LIGHT through LTβR can target a broad range of inflammatory genes in lung fibroblasts, although with some selectivity. We next analyzed whether LIGHT-induced gene transcription was sufficient to result in production of the relevant inflammatory proteins. Interestingly, only IL-6 and GM-CSF were detected in the supernatants of LIGHT-stimulated fibroblasts out of the primary genes that were upregulated (Figures 4F,G), and we could not detect levels of CCL5, CXCL11, TSLP, IL-33, ADAM8, or MMP-9 protein by ELISA (data not shown). The limit of detection of these assays varied from 3.1 to 7.8 pg/ml. It is possible we could not detect protein as it was made at very low levels below the detection limit, or a period of time longer than 3 days was required for these products to be secreted. However, as IL-6 and GM-CSF were found, and their gene transcription was stronger, we favor the notion that transcription of the other genes was not sufficient for promoting detectable levels of protein. This is supported by further experiments described below. These data suggest that although LIGHT can positively affect transcription of a number of inflammatory genes, acting alone it may primarily promote production of the inflammatory proteins ICAM-1, VCAM-1, IL-6, and GM-CSF.

### IL-13 Modulates LIGHT-Induced Activities in Lung Fibroblasts

Because of the restricted induction of inflammatory proteins by LIGHT, this suggested that additional cofactors might be required to enhance the transcription of the other LIGHT-targeted genes to levels sufficient for translation into significant amounts of protein. We previously found that LIGHT could promote IL-13 production from eosinophils, and that IL-13 played an important role in aspects of the lung remodeling response *in vivo* that was dependent on LIGHT (11, 12). As lung fibroblasts express functional IL-4/IL-13 receptors, and IL-13 has been reported to exert some activities that might overlap with those we found for LIGHT (5, 6), we asked if IL-13 could reproduce some of the LIGHT activities in HLF. We also tested if IL-13 could synergize with LIGHT to promote certain inflammatory mediators. We confirmed the expression of IL-4Ralpha and IL-13Ralpha1, the receptors for IL-13, in cultured HLF by flow cytometry and found no change in their levels after LIGHT stimulation (data not shown).

**Figure 5:**
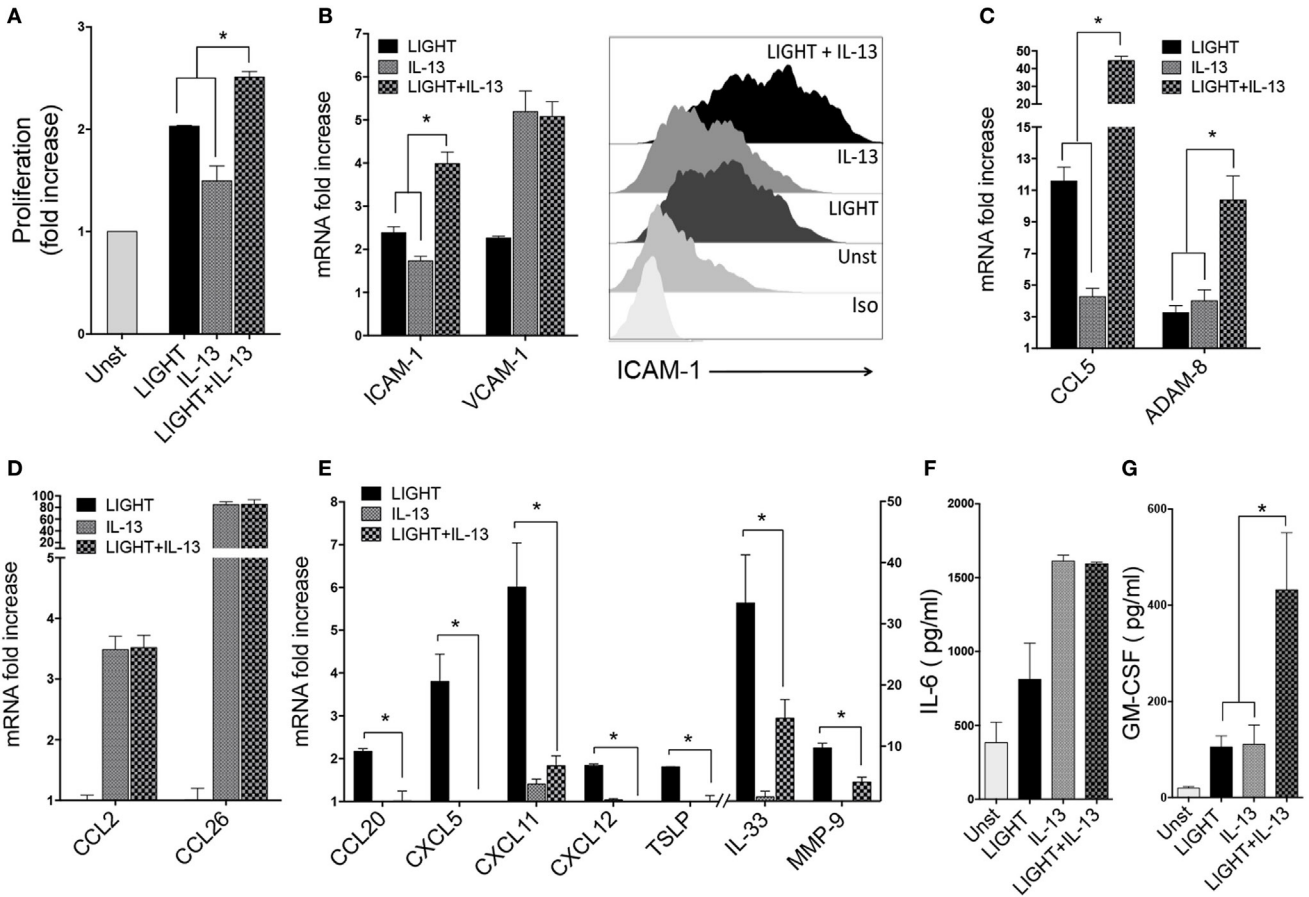
Homologous to Lymphotoxin, exhibits Inducible expression and competes with HSV Glycoprotein D for binding to HVEM, a receptor expressed on T lymphocytes (LIGHT) and IL-13 have distinct as well as shared activities in lung fibroblasts. Human lung fibroblasts (HLFs) were stimulated for 48 h with soluble LIGHT (50 ng/ml, black bars), IL-13 (25 ng/ml, gray bars), or both cytokines (hatched bars). **(A)** Proliferation assessed by thymidine incorporation. **(B)** ICAM-1 and VCAM-1 mRNA expression (left), and flow cytometry of ICAM-1 protein (right). **(C)** mRNA for molecules induced by both LIGHT and IL-13. **(D)** mRNA for molecules only upregulated by IL-13. **(E)** mRNA for molecules only upregulated by LIGHT. All data are mean ± SEM from at least three replicate cultures from three independent experiments, displayed as fold increase above unstimulated. **(F,G)** Mean IL-6 **(F)** and GM-CSF **(G)** protein levels in supernatants after 3 days of stimulation. *n* = 5 independent experiments.

First, we assessed whether IL-13 could promote division of HLF similar to LIGHT, or have any effect on alpha smooth muscle actin expression. As shown in Figure 5A, soluble IL-13 alone moderately increased HLF division, although not to statistically significant levels over that seen with unstimulated cells. Interestingly, simultaneous stimulation of HLF cells with LIGHT and IL-13 significantly enhanced proliferation of HLF cells compared with that seen with LIGHT or IL-13 alone. No change in the expression of HVEM or LTβR was observed after IL-13 stimulation (data not shown). Thus, both LIGHT and IL-13 can influence lung fibroblast proliferation, either at independent times or together. Some reports have also suggested that IL-13 can enhance alpha smooth muscle actin expression in lung fibroblasts (18, 19), while others have not found such an activity (20). We did not detect any effect of IL-13 alone, or in combination with LIGHT, in promoting the induction of alpha smooth muscle actin (data not shown).

Enhancement of gene transcription for the adhesion molecules ICAM-1 and VCAM-1 was also a shared feature of IL-13. It had a lesser effect on ICAM-1 mRNA than LIGHT, but together the two molecules resulted in enhanced ICAM-1 transcript levels compared to either molecule alone. VCAM-1 mRNA was induced by IL-13 alone to a greater extent compared to LIGHT, but no further expression resulted after stimulation with both molecules (Figure 5B). Flow cytometry analysis of these proteins revealed the same pattern as mRNA with LIGHT in combination with IL-13 resulting in higher levels of ICAM-1 (Figure 5B) but not VCAM-1 (data not shown).

We found divergent effects on the other LIGHT-regulated genes when HLF were exposed to LIGHT and IL-13 simultaneously. Transcription of CCL5 and ADAM8 was upregulated to lesser or equivalent extents by IL-13 alone compared to LIGHT, but expression of both of these genes was further enhanced when HLF were stimulated with LIGHT together with IL-13, either in additive or synergistic manners (Figure 5C). However, interestingly this still did not result in detection of either protein in the fibroblast supernatants (data not shown), suggesting additional stimulants are required for production of these molecules or alternatively the time point assayed was not sufficiently late for their detection. As described earlier, LIGHT did not have any significant impact on mRNA expression of CCL2 and CCL26 that were induced in HLF by IL-13, and LIGHT when combined with IL-13 did not modulate these IL-13-driven activities (Figure 5D). Similar to LIGHT, IL-13 action was not sufficient to induce secretion of CCL26 to levels above the ELISA detection limit, and stimulation with IL-13 together with LIGHT did not induce detectable protein (not shown). Interestingly, transcription of several inflammatory molecule genes that were induced by LIGHT alone was not significantly affected by IL-13 alone, namely CCL20, CXCL5, CXCL11, CXCL12, TSLP, IL-33, and MMP-9. Moreover, IL-13 partially or fully antagonized the enhancing effect of LIGHT on these genes (Figure 5E).

Of the two detectable proteins in supernatants of LIGHT-stimulated cells, IL-6 was induced at higher levels by IL-13 compared to LIGHT alone, but no further secretion was found when LIGHT was added with IL-13 (Figure 5F). GM-CSF protein was detected in IL-13-stimulated cultures at levels similar to those induced by LIGHT, and IL-13 and LIGHT synergized together for production of this protein (Figure 5G).

**Figure 6:**
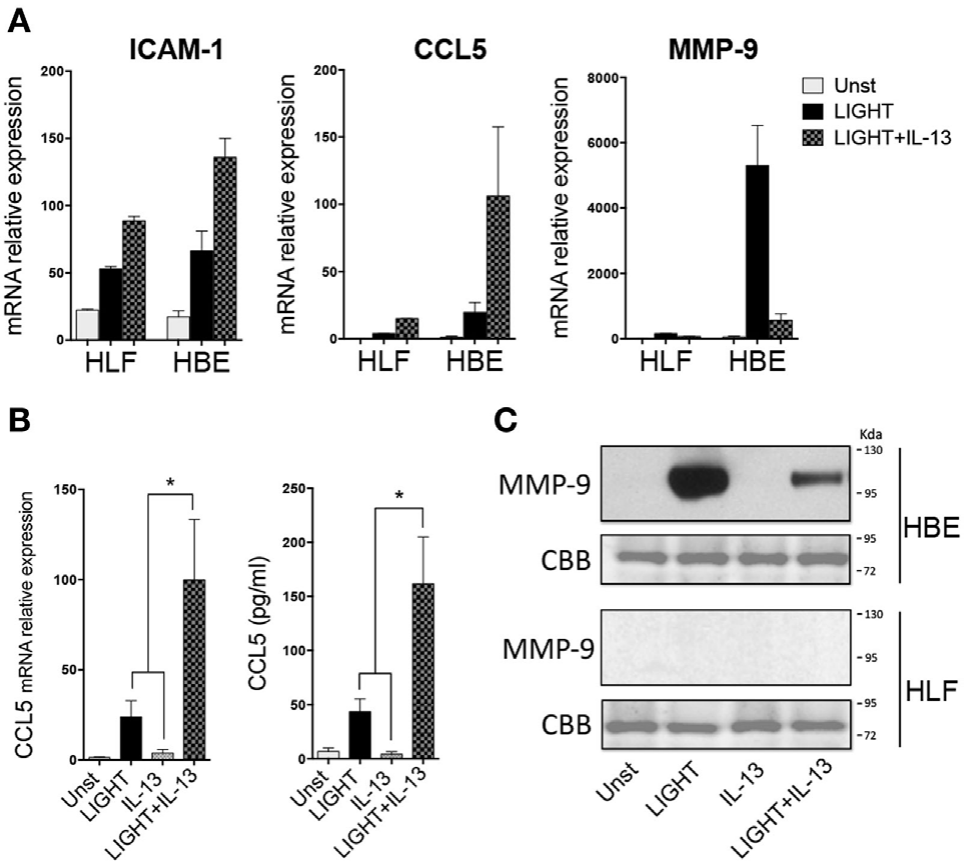
Homologous to Lymphotoxin, exhibits Inducible expression and competes with HSV Glycoprotein D for binding to HVEM, a receptor expressed on T lymphocytes (LIGHT) and IL-13 share similar albeit stronger activities in lung epithelial cells compared to fibroblasts. Human lung fibroblast (HLF) or human bronchial epithelial (HBE) were unstimulated (light gray bars) or stimulated for 48 h with soluble LIGHT (50 ng/ml, black bars), IL-13 (25 ng/ml, gray bars) or both cytokines (hatched bars). **(A)** Relative mRNA expression to housekeeping gene of ICAM-1, CCL5, and MMP-9. **(B)** mRNA or protein levels of CCL5 after 2–3 days of culture of HBE. **(C)** MMP-9 protein in HBE or HLF supernatants after 5 days of culture, by immunoblotting (top). Coomassie Brilliant Blue (CBB) staining of transferred polyacrylamide gel (bottom). Data are mean ± SEM from at least three replicate cultures from three independent experiments, or representative of two experiments.

Lastly, we asked whether the inability of LIGHT or IL-13 to result in production of detectable levels of protein from certain genes, while having an obvious effect on mRNA, might be related to the level of transcription of these genes in HLF. Previously we found that LIGHT could induce mRNA, and production of protein, in primary HBE cells of several of the same molecules we found were targets in HLF (15). We then compared HLF to HBE in their response to LIGHT and IL-13. Significantly, LIGHT induced ICAM-1 mRNA at equivalent levels in HLF and HBE (Figure 6A), correlating with our ability to detect upregulation of ICAM-1 protein in both cell types [see above and Ref. (15)]. However, transcription of CCL5 and MMP-9 induced by LIGHT was markedly higher in HBE compared to HLF. The same pattern of synergy and antagonism, respectively, was observed in both cells when LIGHT was cultured in the presence of IL-13 (Figure 6A). Correlating with this, CCL5 and MMP-9 protein were readily detectable in HBE stimulated with LIGHT, and CCL5 production was further elevated while MMP-9 production was suppressed in the presence of IL-13 (Figures 6B,C). Thus, the action of LIGHT and IL-13 in modulating mRNA for certain genes is highly reproducible and significant, and shared between lung fibroblasts and lung epithelial cells, and the lack of production of significant levels of the protein products in fibroblasts is likely related to the overall level of gene transcription.

## Discussion

We have previously shown in several murine models of lung inflammation, induced by allergens or the fibrosis-inducing antibiotic bleomycin, that the TNF superfamily molecule LIGHT can play a primary role in contributing to features of lung remodeling that have characteristics of severe asthma, IPF, or SSc. Although T cells, macrophages, and eosinophils express one or both of the receptors for LIGHT and are functionally responsive to this cytokine (11, 14), we suggested that the activity of lung structural cells might also be modulated by these receptors. In this regard, we found significant inflammatory effects of LIGHT in bronchial epithelium, including promoting chemokine production and allergic inflammatory mediators such as TSLP (12, 15). Here, we now show that LIGHT has additional actions in HLFs relevant to the pathogenesis of diseases characterized by lung fibrosis and remodeling. Moreover, we show that LIGHT has distinct as well as overlapping activities in lung fibroblasts compared to TGF-β and IL-13, two molecules that are also thought central to tissue inflammation in asthma, IPF, or SSc.

The division and/or differentiation of fibroblasts and their accumulation at the sites of inflammation has long been recognized as a sign of remodeling in the lung tissue. Greater numbers of fibroblasts expressing alpha smooth muscle actin (myofibroblasts) have been seen in the lungs of patients with asthma, IPF, and SSc (21–29). While a number of factors, in particular IL-13 and TGF-β, have been discussed as important for myofibroblast accumulation (5–9, 17–19), we now add LIGHT as a further inflammatory mediator that can promote cell division in lung fibroblasts, similar to its activity in promoting proliferation of T cells (14). We suggest that LIGHT is as potent or more potent in this regard as IL-13, and distinct from TGF-β that antagonized fibroblast proliferation. This activity of LIGHT may not be restricted to fibroblasts from the lung as LIGHT was previously shown to drive proliferation of fibroblast-like synoviocytes from patients with rheumatoid arthritis (30). Interestingly, we did not find that LIGHT or IL-13 had any significant activity in promoting alpha smooth muscle actin expression in lung fibroblasts, unlike TGF-β. This suggests that LIGHT and IL-13 are likely to function upstream, downstream, or independently of TGF-β in controlling the accumulation of myofibroblasts.

The LIGHT also upregulated the expression of the adhesion molecules ICAM-1 and VCAM-1, somewhat similarly to IL-13. This implies that the availability of LIGHT in the lung environment could impact the localization, and possibly maintenance, of myofibroblasts in inflammatory niches of the lungs during phases of remodeling. LIGHT additionally induced the transcription of a select number of CC (5 and 20) or CXC (5, 11, 12) chemokines in lung fibroblasts. These were either distinct from or overlapping with those induced by IL-13, although in the case of the chemokines we could not detect these proteins in supernatants. This implies that LIGHT together with IL-13 could indirectly contribute to the extent of migration and/or retention of T cells, eosinophils, and neutrophils to areas of fibrosis. This would further amplify the activities of the fibroblasts in contributing to lung dysfunction, albeit in cooperation with other undefined cofactors that would be needed to elevate gene transcription in these cells to levels where significant chemokine protein resulted. Interestingly, we previously found that ICAM-1 and VCAM-1 cell surface expression were also upregulated in HBE cells by LIGHT (15), as was transcription of CCL5 and CCL20, and CXCL5 and CXCL11 (15). We showed that LIGHT resulted in secretion of CCL5 protein from bronchial epithelial cells, and here we show this was further enhanced by IL-13. This correlated with a greater induction of mRNA for CCL5 in these cells compared to fibroblasts, although we did not assay for secretion of the other chemokines. Therefore, there are common gene targets of LIGHT in both of these cell types. Alone, or in combination with other inflammatory molecules, LIGHT is then likely to contribute to the ability of fibroblasts that localize adjacent to the bronchial epithelium to participate in the subepithelial inflammatory cell foci that are characteristic of infiltrates seen in lung disease.

The other inflammatory gene targets of LIGHT in lung fibroblasts were GM-CSF and IL-6, IL-33 and TSLP, and MMP-9 and ADAM8. Again, these were either shared activities with IL-13, such as induction of mRNA or protein for IL-6, GM-CSF, and ADAM8, or strikingly distinct from IL-13 in the case of IL-33, TSLP, and MMP-9. As with chemokines, the action of LIGHT was selective in that mRNA for several other similar cytokines or metalloproteinases was not induced. Similarly, we found that gene expression of GM-CSF, IL-6, TSLP, MMP-9, and ADAM8 were promoted in bronchial epithelial cells by LIGHT (12, 15). LIGHT also resulted in IL-6 and GM-CSF protein secretion by fibroblasts and bronchial epithelial cells. We did not detect the other factors in fibroblast supernatants, contrasting with TSLP and MMP-9 protein that were readily detectable in bronchial epithelial cells after LIGHT stimulation (12, 15), and largely correlating with a greater level of gene transcription in the epithelial cells. We did not assess if ADAM8 was secreted by bronchial epithelial cells, but levels of mRNA induced by LIGHT were also greater in these cells compared to lung fibroblasts. Regardless, these results imply that there are a number of targets of LIGHT in structural cells that may be irrespective of the cell type. This might also apply to the origin of the cells, as a prior study in fibroblast-like synoviocytes derived from rheumatoid arthritis patients found that IL-6 protein and mRNA for MMP-9, but not other MMPs, were upregulated by LIGHT (31). Out of the LIGHT-targeted inflammatory mediators, IL-13 only enhanced secretion of IL-6 and GM-CSF, or expression of mRNA for ADAM8, when cultured alone or combined with LIGHT. Moreover, IL-13 antagonized LIGHT driven upregulation of mRNA for TSLP, IL-33, and MMP-9. This again suggests that other as yet undefined inflammatory cofactors may need to act together with LIGHT in lung fibroblasts to result in production of the latter molecules. A similar conclusion was also evident from analysis of mRNA for CCL20, CXCL5, CXCL11, and CXCL12, where IL-13 antagonized the effect of LIGHT on these genes.

GM-CSF and IL-6 can promote the activation and maturation of dendritic cells, macrophages, and T cells, providing an additional mechanism by which lung-expressed LIGHT together with IL-13 could perpetuate lung inflammation. Similarly, our finding that LIGHT can upregulate mRNA expression of IL-33 in particular, and to a lesser extent TSLP, in lung fibroblasts provides another way in which its action could aid a continued inflammatory response in, and around, the airways. The receptors for these cytokines are broadly expressed on T cells, dendritic cells, macrophages, innate lymphoid cells, as well as other cells associated with lung inflammation and remodeling such as eosinophils, mast cells, and neutrophils (32, 33). The metalloproteinases MMP-9 and ADAM8 are both elevated in asthmatic patients (34, 35). They may contribute to lung dysfunction by promoting the release of active proteins within the tissue, regulating cell trafficking to and within the lung, as well as modulating the degradation of extracellular matrix proteins and integrity of the epithelial barrier (36–39). Interestingly, similar to the membrane form of TNF and LTαβ which are cleaved into a soluble form by the metalloproteinase ADAM17, LIGHT has also been proposed to be cleaved from the membrane into a soluble form by metalloproteinase activity. This was shown by blockade of release of LIGHT into the supernatants of activated T cells by a broad-spectrum metalloproteinase inhibitor (40). Although the specific enzyme that targets membrane LIGHT is not known, it is an intriguing possibility that induction of MMP-9 or ADAM8 in fibroblasts or epithelial cells by LIGHT might also function to promote production of the soluble molecule to broaden its reach within the lung tissue as well as the circulation.

Interestingly, and somewhat surprisingly, LTβR but not HVEM was the receptor for LIGHT that controlled the fibroblast activities that we report in this study. This is also in line with the data we previously published on bronchial epithelial cells where LTβR was central to the effects of LIGHT (15). It is also consistent with one study that showed that LTαβ, which only binds LTβR, induced mRNA or protein expression of ICAM-1, and several chemokines including CCL5, in rheumatoid arthritis-derived fibroblast-like synoviocytes (41). Similarly, another study found that LIGHT induction of proliferation of RA joint-derived fibroblast-like synoviocytes, as well as upregulation of ICAM-1 and two chemokines, was inhibited by knockdown of LTβR but not HVEM (30). HVEM was expressed at lower levels than LTβR on lung fibroblasts, as well as the aforementioned synoviocytes, which could have accounted for the findings that it was not active. However, the expression of HVEM on these cells was easily visualized, suggesting that HVEM might still participate in a fibroblast response to LIGHT, but perhaps regulating activities that have not yet been investigated. Future more global gene analysis studies with fibroblasts where LTβR is knocked down will be needed to determine if HVEM is a functional receptor for LIGHT on these cells.

In summary, our results provide more impetus for considering whether therapeutics that block LIGHT activity could be useful for the treatment of severe asthma, IPF, or the interstitial lung disease characteristic of SSc patients. The data imply that therapies that cotarget IL-13 and/or TGF-β together with LIGHT might be considered in those patients with the most severe disease.

## Author Contributions

RA, AM, LM, JT, and MC participated in the design and planning of the study. RA, AM, and LM performed and analyzed experiments. RA, LM, JT, and MC, wrote the article. All authors have read, edited, and approved the manuscript.

## Conflict of Interest Statement

The authors declare that the research was conducted in the absence of any commercial or financial relationships that could be construed as a potential conflict of interest.

## Abbreviations

LIGHT, homologous to Lymphotoxin, exhibits Inducible expression and competes with HSV Glycoprotein D for binding to HVEM, a receptor expressed on T lymphocytes; SSc, systemic sclerosis; IPF, idiopathic pulmonary fibrosis.
