# Supplementary figures and images for: TNFSF14 (LIGHT) Exhibits Inflammatory Activities in Lung Fibroblasts Complementary to IL-13 and TGF-β
Source: Front Immunol. 2018 Mar 19;9:576. doi: 10.3389/fimmu.2018.00576 (PMC5868327; doi:10.3389/fimmu.2018.00576)

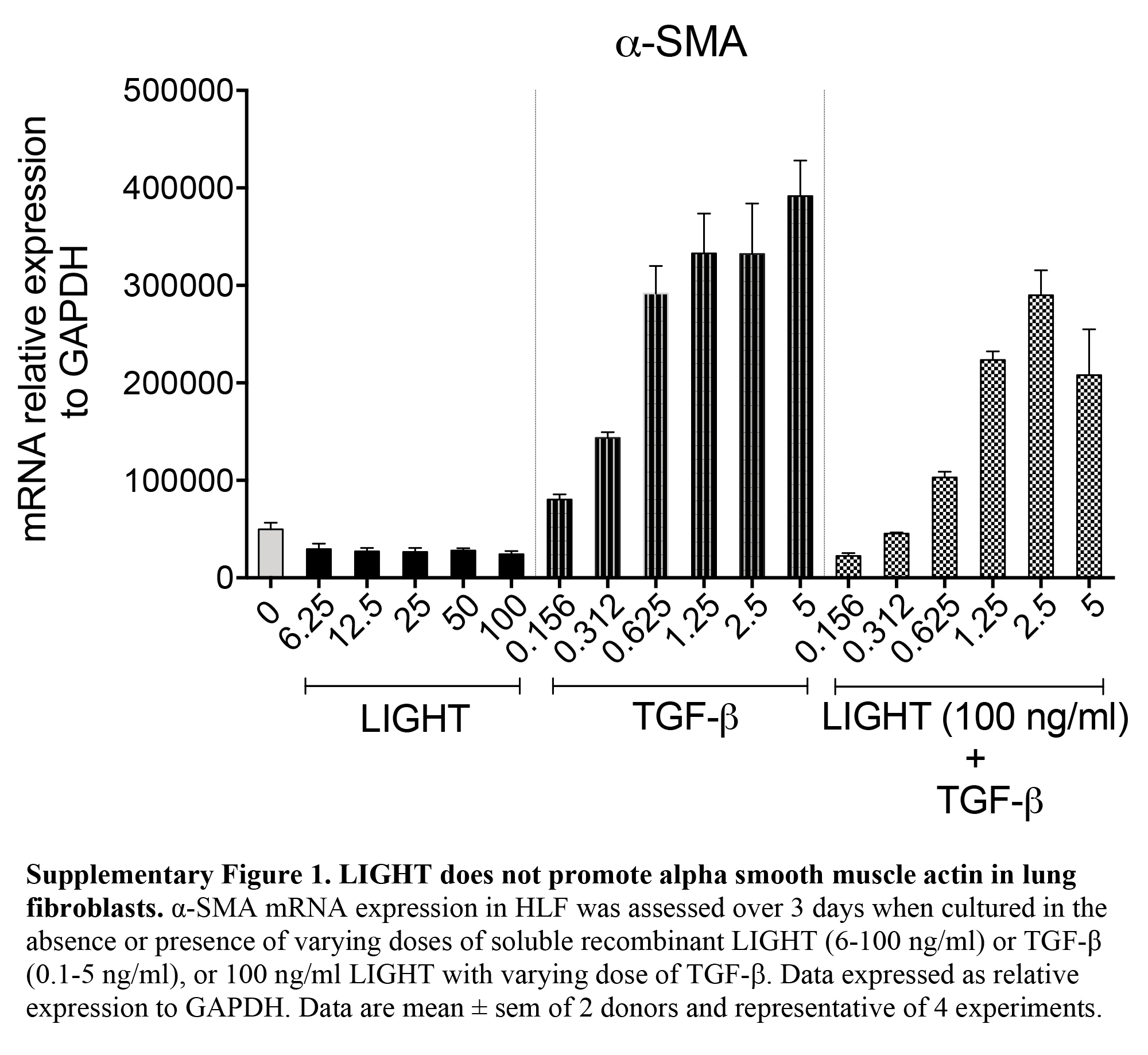

Supplement: Supplementary file 1 [file image_1.jpeg]

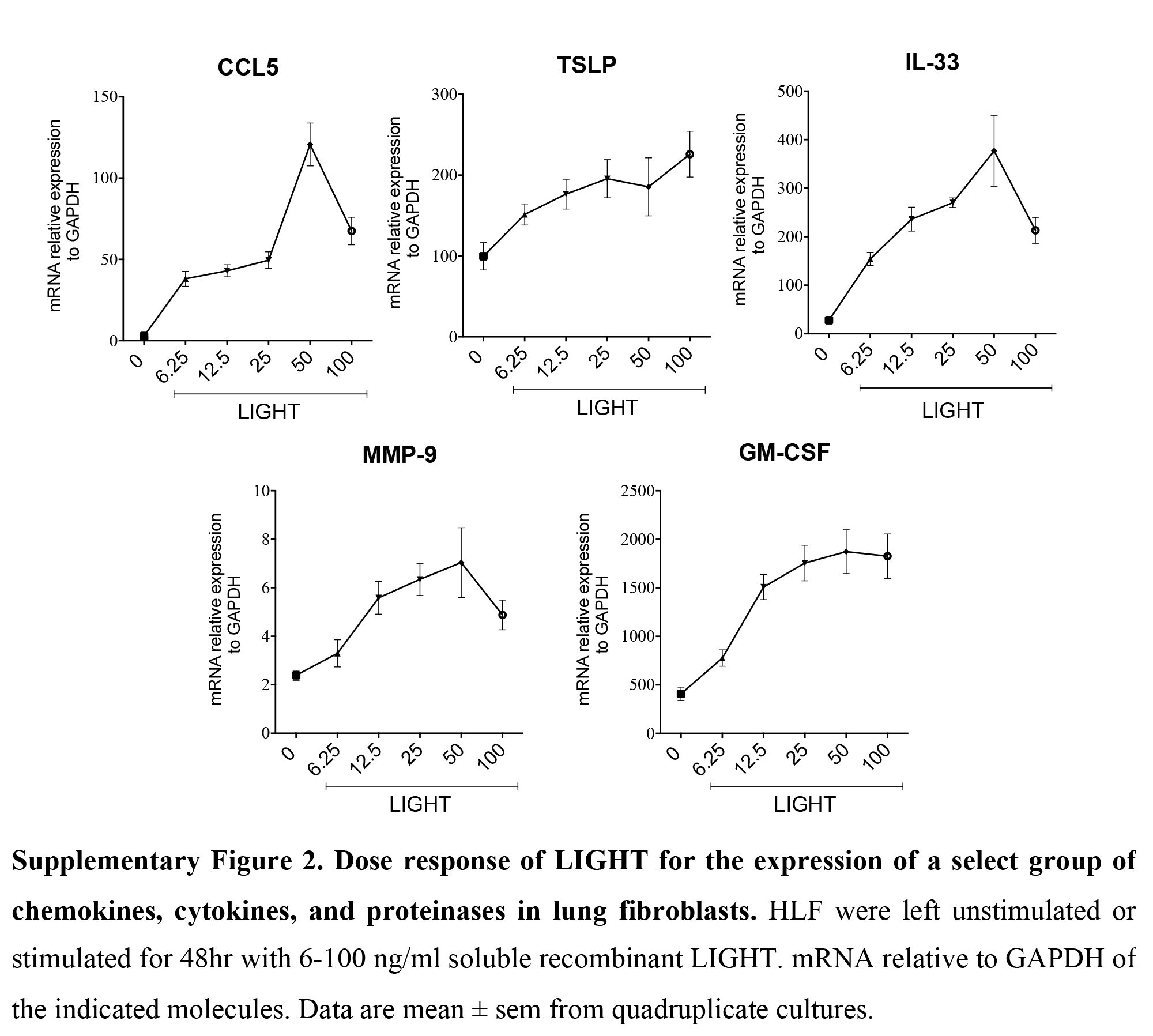

Supplement: Supplementary file 2 [file image_2.jpeg]

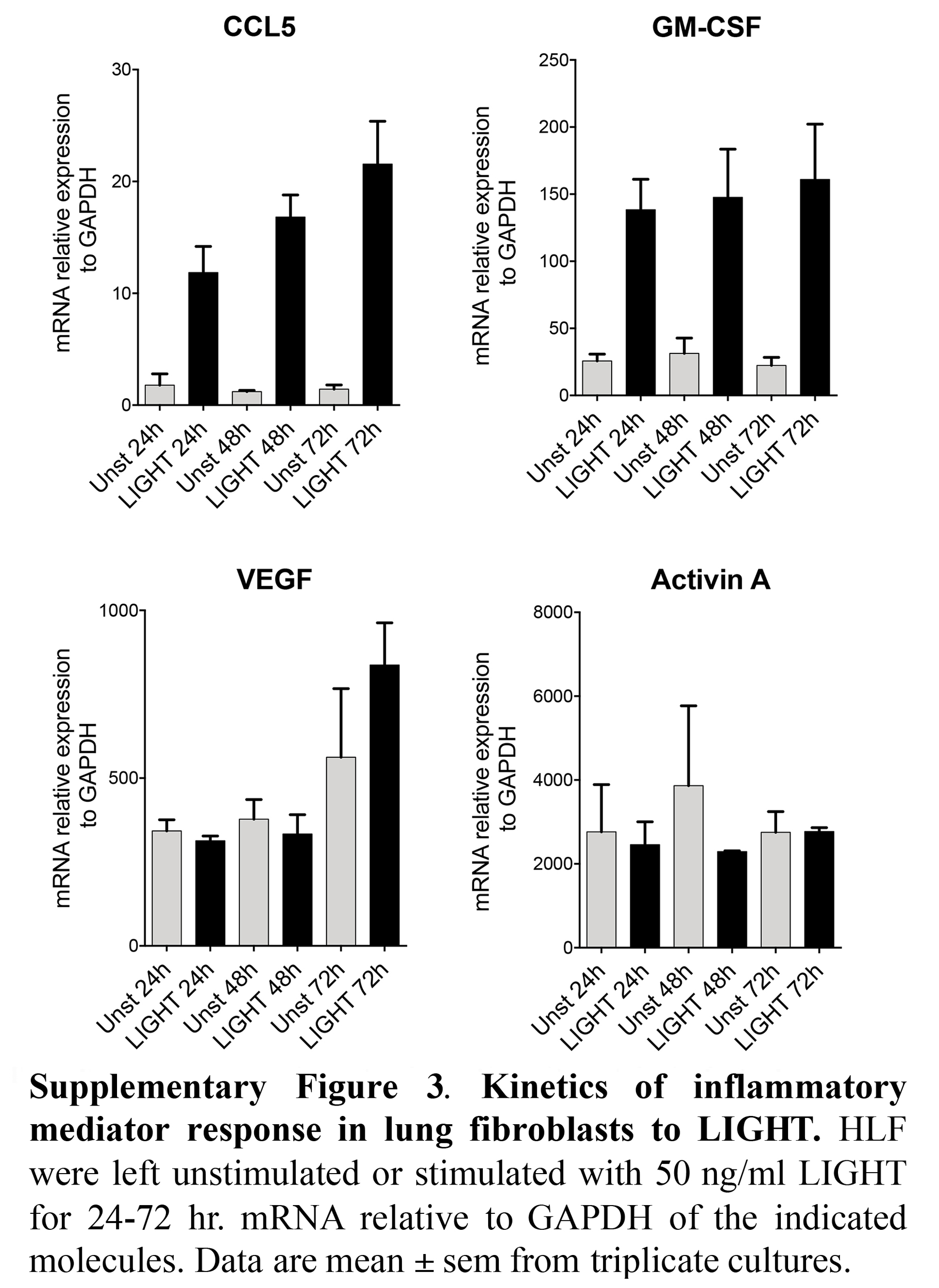

Supplement: Supplementary file 3 [file image_3.jpeg]
